# Comprehensive Molecular Analyses of a TNF Family-Based Gene Signature as a Potentially Novel Prognostic Biomarker for Cervical Cancer

**DOI:** 10.3389/fonc.2022.854615

**Published:** 2022-03-22

**Authors:** Yan Ma, Xiaoyan Zhang, Jiancheng Yang, Yanping Jin, Ying Xu, Jianping Qiu

**Affiliations:** Department of Gynecology and Obstetrics, The Affiliated Suzhou Hospital of Nanjing Medical University, Suzhou Municipal Hospital, Suzhou, China

**Keywords:** cervical cancer, TNF family genes, prognosis, immunotherapy response, TCGA

## Abstract

**Background:**

Increasing evidence suggests that tumour necrosis factor (TNF) family genes play important roles in cervical cancer (CC). However, whether TNF family genes can be used as prognostic biomarkers of CC and the molecular mechanisms of TNF family genes remain unclear.

**Methods:**

A total of 306 CC and 13 normal samples were obtained from The Cancer Genome Atlas (TCGA) and Genotype-Tissue Expression (GTEx) databases. We identified differentially expressed TNF family genes between CC and normal samples and subjected them to univariate Cox regression analysis for selecting prognostic TNF family genes. Least absolute shrinkage and selection operator (LASSO) regression and multivariate Cox regression analyses were performed to screen genes to establish a TNF family gene signature. Gene set enrichment analysis (GSEA) was performed to investigate the biological functions of the TNF family gene signature. Finally, methylation and copy number variation data of CC were used to analyse the potential molecular mechanisms of TNF family genes.

**Results:**

A total of 26 differentially expressed TNF family genes were identified between the CC and normal samples. Next, a TNF family gene signature, including CD27, EDA, TNF, TNFRSF12A, TNFRSF13C, and TNFRSF9 was constructed based on univariate Cox, LASSO, and multivariate Cox regression analyses. The TNF family gene signature was related to age, pathological stages M and N, and could predict patient survival independently of clinical factors. Moreover, KEGG enrichment analysis suggested that the TNF family gene signature was mainly involved in the TGF-β signaling pathway, and the TNF family gene signature could affect the immunotherapy response. Finally, we confirmed that the mRNA expressions of CD27, TNF, TNFRSF12A, TNFRSF13C, and TNFRSF9 were upregulated in CC, while that of EDA was downregulated. The mRNA expressions of CD27, EDA, TNF, TNFRSF12A, TNFRSF13C, and TNFRSF9 might be influenced by gene methylation and copy number variation.

**Conclusion:**

Our study is the first to demonstrate that CD27, EDA, TNF, TNFRSF12A, TNFRSF13C, and TNFRSF9 might be used as prognostic biomarkers of CC and are associated with the immunotherapy response of CC.

## Background

Cervical cancer (CC) is a common gynaecological malignancy. Approximately 570,000 new cases of CC and 311,365 deaths were reported worldwide in 2018, making CC the third most common cancer among women (1). Although improved screening and health literacy have decreased the incidence rates of CC in recent years, CC remains the leading cause of cancer-related deaths among women in developing countries (2). Many risk factors can affect the development of CC, such as high-risk HPV infection, smoking, health status, and economic status (3, 4). CC rates dropped by 1.3% in 2015 compared to that in 2012, given the production of HPV vaccines, improvements in living conditions, and early screening (5). However, although 80% of early CC cases can be treated with surgery, radiotherapy, or chemotherapy, the number of patients in advanced CC stages is high and where the prognosis remains poor (6). Additionally, some patients relapse easily despite undergoing appropriate surgery and chemotherapy (7). Therefore, it is important to screen new biomarkers suitable for CC prognosis to improve the effectiveness of treatment and also develop precise treatment strategies.

The tumour necrosis factor (TNF) and TNF receptor (TNFR) superfamilies (TNFSF/TNFRSF) include 19 ligands and 29 receptors (8). A previous study has shown that communication pathways mediated by TNFSF/TNFRSF members can regulate inflammation and control cell death, proliferation, and differentiation (8). TNFSF/TNFRSF members exert proinflammatory effects by activating the nuclear factor NF-κB pathway, which is majorly involved in protection against pathogens and cancer (9). Inflammation can also be conducive to tumour proliferation, metastasis, and angiogenesis in many types of cancer (10). In addition, TNF-induced apoptosis has high potential in anticancer therapy. For example, the TNF-related apoptosis-inducing ligand (TRAIL), a member of the TNF family (11), can selectively induce cancer cell apoptosis by binding or trimerising their functional receptors (12). In CC, all TRAIL receptors are expressed in both normal cervical epithelial cells and tumour cells. Moreover, the TRAIL receptors TRAIL-R1 and -R2 are highly expressed in tumour cells than in normal epithelial cells, and their expression is associated with CC cell apoptosis (13). Another study showed that melatonin enhances TNF-α-induced mitochondrial apoptosis in HeLa cells by inactivating the CaMKII/Parkin/mitophagy axis. TNF-α-induced lncRNA LOC105374902 may act as a ceRNA of miR-1285-3p to promote the expression of RPL14 as well as the migration, invasion, and epithelial-mesenchymal transition (EMT) of CC cells (14). TNF family genes have a significant impact on the occurrence and development of tumours (15); these can be used as prognostic markers of tumours and can affect the immunotherapy response of tumours (16). However, the role of TNF family genes in CC remains unclear, and their potential functional role in the prognosis of cancer should be studied further.

In this study, we downloaded transcriptomic data of CC and matched normal samples from The Cancer Genome Atlas (TCGA, https://portal.gdc.cancer.gov/). The transcriptomic data of normal cervical samples were downloaded from Genotype-Tissue Expression (GTEx) database (https://commonfund.nih.gov/gtex). First, we sought to identify differentially expressed TNF family genes in cervical tumours versus normal cervical tissues. The TNF family gene signal was established based on univariate and multivariate Cox and LASSO regression analyses assess CC prognosis. In addition, we further analysed the molecular mechanism by which TNF family gene signaling affects immunotherapy and gene alterations in patients. Finally, we analysed the mRNA expression levels of risk model genes using qRT-PCR. This study can help facilitate the prognosis of CC and the development of phasic immunotherapy strategies.

## Methods

### Data Selection

RNA-seq data [fragments per kilobase of exon model per million mapped fragments (FPKM)] of 306 CC and three matched normal cervical samples, copy number variation data of 297 CC samples, DNA methylation data of 312 CC samples, and clinical information (including overall survival information) data of 306 patients with CC in TCGA database were acquired from the Cancer Genomics Browser of the University of California Santa Cruz (UCSC) Xena database (https://xenabrowser.net). After excluding samples without survival information, 293 samples with survival information were used for survival analysis, and their detailed clinical information was shown in **Table 1**. Moreover, the RNA-seq of 10 normal cervical samples were acquired from the GTEx database (17). Furthermore, the GSE44001 dataset, including 300 CC patients with survival information, was downloaded from the Gene Expression Omnibus (GEO) database (https://www.ncbi.nlm.nih.gov/geo/) and acted as a testing set. The 47 TNF family genes were obtained from Zhang et al. (16). Finally, the RNA-seq data of 1,082 breast cancer (BRCA), 378 ovarian cancer (OV), 544 uterine corpus endometrial carcinoma (UCEC), and 54 uterine carcinosarcoma (UCS) samples with overall survival information were analysed to further investigate the role of TNF family genes in gynaecologic tumours as well as in BRCA.

**Table 1:** The clinical information of these 293 CC patients with survival information in the TCGA databse.

Characteristics	No.
Total Cases	293
Age (<50)	176
Age (>=50)	111
clinical_stage (Stage I)	159
clinical_stage (Stage II)	64
clinical_stage (Stage III)	81
clinical_stage (Stage IV)	22
neoplasm_histologic_grade (G1)	19
neoplasm_histologic_grade (G2)	129
neoplasm_histologic_grade (G3)	118
neoplasm_histologic_grade (G4)	1
neoplasm_histologic_grade (GX)	24
new_tumor_event_after_initial_treatment (NO)	15
new_tumor_event_after_initial_treatment (YES)	12
pathologic_M (M0)	107
pathologic_M (M1)	11
pathologic_M (MX)	127
pathologic_N (N0)	129
pathologic_N (N1)	56
pathologic_N (N0)	65
pathologic_T (T1)	137
pathologic_T (T2)	68
pathologic_T (T3)	16
pathologic_T (T4)	11
pathologic_T (TX)	18

### Identification of Differentially Expressed TNF Family Genes

The limma package in R was used to eliminate batch benefits between CC and three matched normal cervical samples from TCGA and 10 normal cervical samples from the GTEx database and to identify the differentially expressed genes (DEGs) between CC and normal samples (18). Differentially expressed TNF family genes were identified by overlapping DEGs and TNF family genes. Box plots and heatmaps were plotted to present the expression levels of differentially expressed TNF family genes using the ggplot2 and pheatmap packages in R, respectively.

### Construction and Validation of TNF Family Gene Signature

After excluding patients with CC for whom overall survival information was not available, 293 patients with CC were selected to construct a TNF family gene signature to predict the overall survival of patients with CC. To ensure the validity of the TNF family gene signature, the entire set of 293 patients with CC was randomly divided into a training set (205 patients with CC) and a validation set (88 patients with CC) according to a 7:3 ratio. First, univariate Cox regression analysis was used to identify genes related to the overall survival of patients with CC from differentially expressed TNF family genes in the training set (P < 0.05). Next, least absolute shrinkage and selection operator (LASSO) regression analysis based on the glmnet package in R were conducted to determine the best combination of genes for constructing a TNF family gene signature in the training set (19). Then, the “step” function in R was used to perform a multivariate Cox regression analysis to construct the optimal TNF family gene signature. In addition, the TNF family gene signature was established using gene expression values and the corresponding Cox coefficient. The algorithm of the risk score value for one patient with CC was as follows: (gene 1 expression × gene 1 coefficient) + (gene 2 expression × gene 2 coefficient) + … + (gene n expression × gene n coefficient) (20). The risk scores of patients with CC in the training set, validation set, and entire set were calculated using the aforementioned algorithm. patients with CC in the training, validation, and entire sets were divided into high- and low-risk groups based on the optimal threshold of risk scores. Finally, the Kaplan-Meier (KM) and time-dependent receiver operating characteristic (ROC) curves were used to assess the validity of the TNF family gene signature.

### Association Between TNF Family Gene Signature and Clinical Characteristics

The association between the TNF family gene signature and clinical characteristics of patients with CC in the entire set, including radiation therapy, race, pathological grade, pathological M, N, and T stages, age, and histologic grade, were investigated using either one-way ANOVA or the Wilcoxon test.

### Construction and Evaluation of a TNF Family Gene Signature-Related Nomogram

The TNF family gene signature and clinical characteristics, including radiation therapy, race, pathological grade, pathological M, N, and T stages, age, and histologic grade, were used to screen independent prognostic factors by performing univariate and multivariate Cox regression analyses in the entire set. Next, a nomogram based on independent prognostic factors identified using multivariate Cox regression analysis was established using the rms package in R (21). Calibration curves were used to observe the predictive power of the nomogram.

### Functional Analysis of TNF Family Gene Signature

Gene set enrichment analysis (GSEA) was used to investigate the dysregulated Kyoto encyclopedia of genes and genomes (KEGG) signaling pathways between the high- and low-risk groups based on all of their gene expression matrices using the clusterProfiler package in R (22). The gene sets c2.cp.kegg.v7.4.symbols.gmt and c5.go.v7.4.symbols.gmt were selected as reference gene sets, and NOM P < 0.05 was considered significant.

### Correlation Between TNF Family Gene Signature and Immunotherapy Response

To explore whether the TNF family gene signature could predict immunotherapy response, Cell-type Identification By Estimating Relative Subsets Of RNA Transcripts (CIBERSORT) algorithm was firstly used to characterize 22 immune cell composition of the patients with CC in the high- and low-risk groups (23). Next, the expression levels of immune checkpoint molecules were compared between the high- and low-risk groups using the Wilcoxon test. Tumour immune dysfunction and exclusion (TIDE) scores of the high- and low-risk groups were calculated and compared to explore whether the gene signature predicts the immunotherapy response (24). Subclass mapping was used to determine the immunotherapy response (25).

### Potential Regulatory Mechanisms of Genes in TNF Family Gene Signature

To explore the potential regulatory mechanisms of genes in the TNF family gene signature, the methylation levels of genes in the TNF family gene signature were first investigated using the ChAMP package in R. The copy number variations of genes in the TNF family gene signature were analysed, and the potential miRNAs of genes in this signature were predicted using the miRDB and miRwalk databases.

### Prognostic Significance of the TNF Family Gene Signature in Gynaecologic Tumours and BRCA

To investigate whether genes in the TNF family gene signature could affect the prognosis of other gynaecologic tumours and BRCA, univariate Cox regression analysis based on the overall survival information and expression of genes in the TNF family gene signature was performed.

### Quantitative Real-Time PCR

To investigate the mRNA expression of genes in the TNF family gene signature, seven patients with CC were underwent treatment at Suzhou Municipal Hospital were included in this study, and carcinoma and paracancerous tissue samples were collected from each patient to perform quantitative real-time PCR. Informed consent was obtained from all participating individuals, and all steps involving human subjects were approved by the ethics committee of Suzhou Municipal Hospital.

Total RNA of 14 matched tissues from the seven patients with CC was extracted using the traditional TRIzol-based method. The extracted RNA was reverse-transcribed into complementary DNA (cDNA) using a SureScript First-Strand cDNA Synthesis Kit (Xavier Corporation, Guangzhou, China), according to the manufacturer’s instructions. Quantitative real-time PCR was performed using a 2720 Thermal Cycler General PCR instrument (Applied Biosystems, Inc., Carlsbad, CA, USA) and a CFX96 real-time quantitative fluorescence thermal cycler (Bio-Rad, Hercules, CA, USA). The 2^-11ΔΔCt^ method was used to calculate the relative expression of genes with internal reference *GAPDH*. The primer sequences are listed in **Table S1**.

### Statistical Analysis

Data analyses were performed based on the R version 4.1.1. The log-rank test was used to test the difference in overall survival between the high- and low-risk groups. Unless otherwise stated, statistical significance was set at P < 0.05.

## Results

### Identification of Differentially Expressed TNF Family Genes

**Figure 1:**
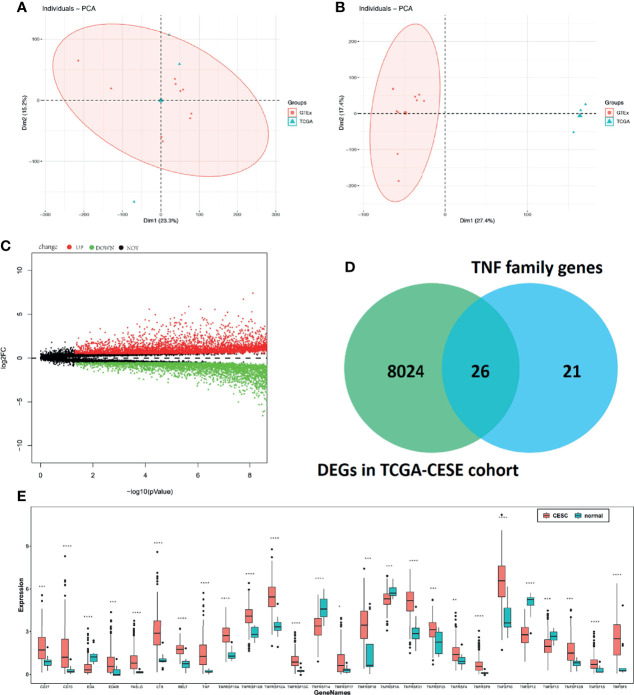
Molecular characteristics and expression profile of tumour necrosis factor (TNF) family members in cervical cancer. Principal component analysis (PCA) before **(A)** and after **(B)** batch effect treatment of TCGA and GTEx samples. **(C)** Volcano plots showing the number of differentially expressed genes. Red dots represent upregulated genes, and green dots represent downregulated genes. **(D)** Venn diagram showing the intersections of genes between TCGA data and TNF family genes. **(E)** Boxplots show differences in the expression levels of TNF family members. *means p < 0.05; **means p < 0.01; ***means p < 0.001; ****means p < 0.0001.

Considering the heterogeneity of TCGA and GTEx samples, we first performed a batch effect treatment. The 306 CC and 13 normal samples that were processed met the criteria for further analysis (**Figures 1A, B**). With a cut-off value of P < 0.05 and |log2FC| > 0.5, 8,050 DEGs were screened between CC and normal samples, including 4,416 upregulated and 3,634 downregulated ones (**Figure 1C**). After overlapping with 47 TNF family genes, 26 differentially expressed TNF family genes were identified (**Figure 1D**); their expression levels are shown in **Figure 1E**.

### Construction and Validation of a TNF Family Gene Signature

**Figure 2:**
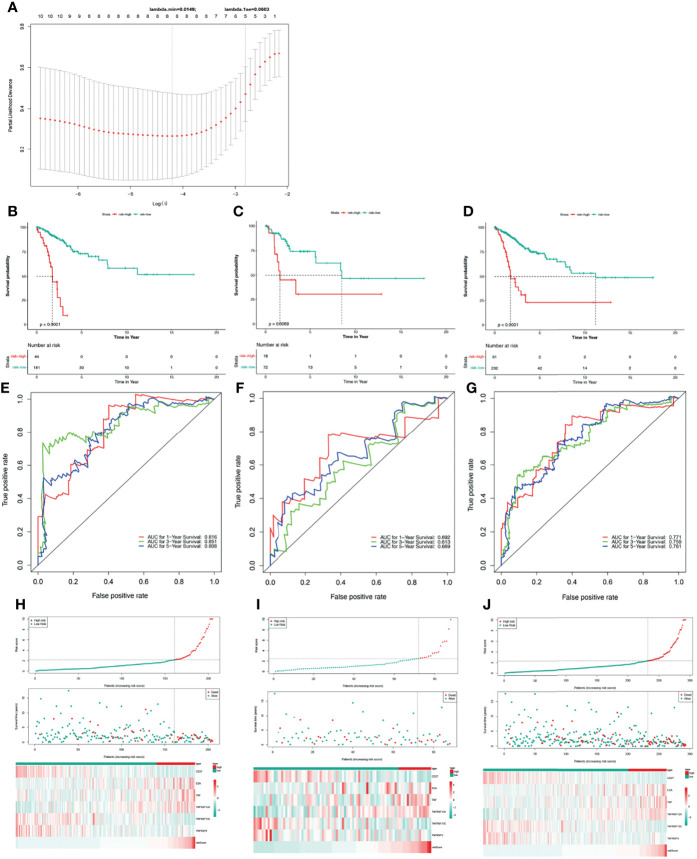
Construction and validation of a TNF family gene signature. **(A)** The most proper log (lambda) value in the LASSO model. **(B)** The Kaplan-Meier curve of the OS for patients with CC based on the risk score for the training set. **(C)** The Kaplan-Meier curve of the OS for patients with CC based on the risk score for the testing set. **(D)** The Kaplan-Meier curve of the OS for patients with CC based on the risk score for the entire set. **(E)** Time-dependent ROC curves of the risk score for the 1-, 3-, and 5-year OS rate prediction for the training set. **(F)** Time-dependent ROC curves of the risk score for the 1-, 3-, and 5-year OS rate prediction for the testing set. **(G)** Time-dependent ROC curves of the risk score for the 1-, 3-, and 5-year OS rate prediction for the entire set. **(H)** Distribution of the risk score, survival status, and gene expression panel for the training set. **(I)** Distribution of the risk score, survival status, and gene expression panel for the testing set. **(J)** Distribution of the risk score, survival status, and gene expression panel for the entire set.

Based on univariate Cox regression analysis of the training set, the 10 differentially expressed TNF family genes *CD27*, *EDA*, *FASLG*, *TNF*, *TNFRSF10B*, *TNFRSF12A*, *TNFRSF13C*, *TNFRSF14*, *TNFRSF9*, and *TNFSF13B* were found to be associated with CC prognosis (P < 0.05, **Table 2**). Subsequently, *CD27*, *EDA*, *TNF*, *TNFRSF10B*, *TNFRSF12A*, *TNFRSF13C*, *TNFRSF9*, and *TNFSF13B* were preserved by LASSO regression analysis and used for multivariate Cox regression analysis (**Figure 2A**). In addition, the expression levels and regression coefficients of *CD27*, *EDA*, *TNF*, *TNFRSF12A*, *TNFRSF13C*, and *TNFRSF9* were used to construct a TNF family gene signature through multivariate Cox regression analysis (**Table 3**). *CD27*, *TNFRSF13C*, and *TNFRSF9* were found protective factors (hazard ratio [HR] < 1), while *EDA*, *TNF*, and *TNFRSF12A* as risk factors (HR > 1) (**Table 3**). The risk score of each patient was calculated as follows: risk score = (−0.370) × *CD27* expression + 0.579 × *EDA* expression + 0.400 × *TNF* expression +(0.403) × *TNFRSF12A* expression + (−0.582) × *TNFRSF13C* expression + (−0.682) × *TNFRSF9* expression (**Table 3**).

**Table 2:** Identification of 10 TNF family genes by univariate Cox regression analysis.

Gene	HR	Lower 95% CI	Upper 95% CI	p value
CD27	0.58	0.421	0.798	0.000838
EDA	2.016	1.222	3.327	0.00606
FASLG	0.638	0.423	0.96	0.031296
TNF	1.489	1.118	1.982	0.00646
TNFRSF10B	1.63	1.064	2.498	0.024742
TNFRSF12A	1.541	1.159	2.05	0.002971
TNFRSF13C	0.317	0.153	0.656	0.001956
TNFRSF14	0.675	0.479	0.95	0.024258
TNFRSF9	0.449	0.233	0.863	0.016397
TNFSF13B	0.619	0.438	0.874	0.00642

HR, hazard ratio; CI, Confidence interval.

**Table 3:** TNF family gene signature constructed with six TNF family genes using multivariate Cox regression analysis.

Gene	HR	HR.95L	HR.95H	P value	Cox Coefficient
CD27	0.690594	0.448524	1.06331	0.092726	-0.370
EDA	1.784758	1.058791	3.008489	0.029675	0.579
TNF	1.491831	1.13535	1.960239	0.00409	0.400
TNFRSF12A	1.496109	1.112881	2.011306	0.007623	0.403
TNFRSF13C	0.558982	0.283221	1.103241	0.093596	-0.582
TNFRSF9	0.505494	0.211322	1.20917	0.125244	-0.682

Thus, we stratified patients with CC in the training set into low- and high-risk groups based on the optimum threshold of the risk score. Interestingly, we found that the overall survival of patients in the low-risk group was clearly better than that of patients in the high-risk group (**Figure 2B**), and patients in the low-risk group had longer survival times than those in the high-risk group (**Figure 2H**). ROC analysis revealed that the areas under curves (AUCs) used to predict the 1-, 3-, and 5-year survival rates of patients with CC were 0.816, 0.851, and 0.808, respectively (**Figure 2E**). *CD27*, *TNFRSF13C*, and *TNFRSF9* showed higher expression in the low-risk group than in the high-risk group. However, the other three genes exhibited lower expression in the low-risk group than in the high-risk group (**Figure 2H**), which was consistent with the results of multivariate Cox regression analysis (**Table 3**). Finally, we assessed the predictive capabilities of the TNF family gene signature in the validation, testing, and entire sets. The KM analyses in the validation and entire sets yielded results consistent with those of the training set; namely, the low-risk patients were associated with better prognosis (**Figures 2C, D**). The ROC curves of both the validation and entire sets also suggested that the TNF family gene signature is a good prognostic factor of CC (**Figures 2F, G**). Similarly, *CD27*, *TNFRSF13C*, and *TNFRSF9* also showed higher expression in the low-risk group than in the high-risk group. However, the other three genes exhibited lower expression in the low-risk group than in the high-risk group (**Figures 2I, J**). Notably, the KM curve of the testing set revealed that the high-risk patients were related to poor prognosis, the ROC curve of the testing set showed that the TNF family gene signature did not have good predictive power (**Figure S1**).

### Relationship Between TNF Family Gene Signature and Clinical Characteristics

**Figure 3:**
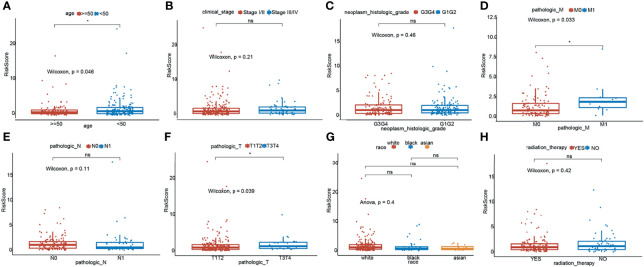
Relationship of risk score and clinicopathological characteristics of CC patients. Distribution of the risk scores in different cohorts stratified by the subtype of age **(A)**, clinical stage **(B)**, neoplasm histologic grade **(C)**, pathological M stage **(D)**, pathological N stage **(E)**, pathological T stage **(F)**, race **(G)** and radition therapy **(H)**. *means p < 0.05; ns represents no significance.

To investigate the relationship between the TNF family gene signature and clinical characteristics, we compared the risk scores between different clinical characteristics across the entire set. As illustrated in **Figure 3A**, patients younger than 50 years had higher risk scores. Moreover, M1 patients showed higher risk scores than M0 patients (**Figure 3D**), and T3/T4 patients exhibited higher risk scores than T1/T2 patients (**Figure 3E**). However, the TNF family gene signature was not related to any other clinical characteristic (**Figure 3**).

### Construction of a TNF Family Gene Signature-Related Nomogram

**Figure 4:**
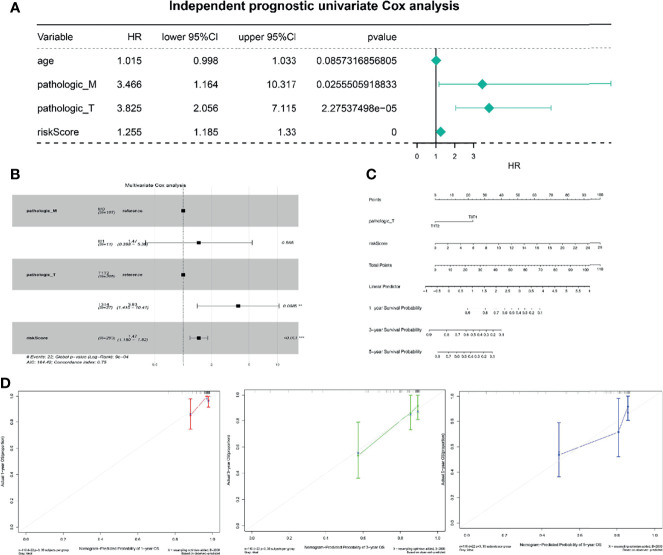
Independent prognostic analysis of risk scores. **(A)** Four factors were included in the univariate Cox analysis, and three of them were significant. HR is the risk ratio, and the lower/upper 95% CI is the 95% confidence interval of the risk value. **(B)** Multivariate analysis results show that T staging and risk score are significant. **(C)** The nomogram shows that T staging and risk score have significant influence on the prognostic survival time of CC patients. **(D)** Calibration curve suggesting that the nomogram holds implications in the prognosis of CC.

To better use the TNF family gene signature, we aimed to construct a nomogram by integrating all TNF family gene signatures and clinical characteristics in the entire set. Based on univariate Cox regression analysis, pathological M and T stages and risk score were found to be associated with CC prognosis (**Figure 4A**), which were the only independent factors for patients with CC (**Figure 4B**). A nomogram integrating pathological T stage and risk score was constructed to predict the 1-, 3-, and 5-year overall survival of patients with CC (**Figure 4C**). The calibration curves suggested that the nomogram could predict CC onset owing to good agreement between the predictions and observations (**Figure 4D**).

### Functional Enrichment of the TNF Family Gene Signature

To further explore the potential functions of the TNF family gene signature, GSEA was performed to observe the potential KEGG signaling pathway related to the TNF family gene signature in the entire set. As is shown in **Table 4**, genes in the high-risk group were mainly involved in the galactose metabolism, N glycan biosynthesis, O glycan biosynthesis, purine metabolism, and TGF-β signaling pathways. Moreover, we found that genes in the low-risk group were mainly involved in the immune-related pathways, such as B cell receptor signaling pathway, primary immunodeficiency pathway, intestinal immune network for IgA production, T cell receptor signaling pathway, and chemokine signaling pathway (**Table 5**). Thus, the TNF family gene signature might affect prognosis of CC mainly through regulating of immune, metabolic and tumor-related pathways.

**Table 4:** The significantly enriched KEGG pathways based on GSEA results (P < 0.01, FDR < 0.25).

NAME	SIZE	ES	NES	NOM p-val
KEGG_GALACTOSE_METABOLISM	25	0.570	1.751	0.011
KEGG_N_GLYCAN_BIOSYNTHESIS	46	0.535	1.786	0.016
KEGG_GLYCOSAMINOGLYCAN_BIOSYNTHESIS_CHONDROITIN_SULFATE	22	0.655	1.716	0.020
KEGG_O_GLYCAN_BIOSYNTHESIS	30	0.580	1.644	0.021
KEGG_HYPERTROPHIC_CARDIOMYOPATHY_HCM	83	0.459	1.567	0.030
KEGG_PURINE_METABOLISM	159	0.315	1.441	0.031
KEGG_FOCAL_ADHESION	199	0.496	1.662	0.034
KEGG_TGF_BETA_SIGNALING_PATHWAY	86	0.456	1.565	0.040
KEGG_AXON_GUIDANCE	129	0.393	1.476	0.042

**Table 5:** The top10 KEGG pathway significantly enriched in the low-risk group.

NAME	SIZE	ES	NES	NOM p-val
KEGG_B_CELL_RECEPTOR_SIGNALING_PATHWAY	75.00	-0.64	-2.09	0.00
KEGG_PRIMARY_IMMUNODEFICIENCY	35.00	-0.88	-2.00	0.00
KEGG_INTESTINAL_IMMUNE_NETWORK_FOR_IGA_PRODUCTION	46.00	-0.80	-1.91	0.00
KEGG_T_CELL_RECEPTOR_SIGNALING_PATHWAY	108.00	-0.60	-1.94	0.00
KEGG_ARACHIDONIC_ACID_METABOLISM	58.00	-0.55	-1.77	0.00
KEGG_CHEMOKINE_SIGNALING_PATHWAY	188.00	-0.55	-1.89	0.00
KEGG_ALDOSTERONE_REGULATED_SODIUM_REABSORPTION	42.00	-0.54	-1.71	0.01
KEGG_FC_GAMMA_R_MEDIATED_PHAGOCYTOSIS	96.00	-0.48	-1.78	0.01
KEGG_FC_EPSILON_RI_SIGNALING_PATHWAY	79.00	-0.46	-1.69	0.01
KEGG_AUTOIMMUNE_THYROID_DISEASE	50.00	-0.72	-1.82	0.01

### Correlation Between the TNF Family Gene Signature and Immunotherapy Response

**Figure 5:**
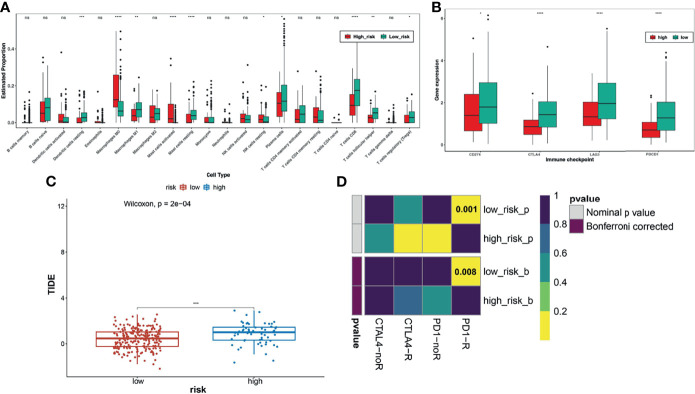
Analysis of immune checkpoint differences in high- and low-risk groups. **(A)** The differences in immune cell infiltration between high and low risk groups. **(B)** Expression levels of immune checkpoint molecules between the high-risk and low-risk groups. **(C)** TIDE scores significantly differ between the high- and low-risk groups, suggesting a higher probability of immune escape in the high-risk group. **(D)** The low-risk group is more likely to be more sensitive to anti-PD1 therapy. *FDR < 0.05; **means p < 0.01; ***means p < 0.001; ****means p < 0.0001. ns represents no significance.

To further explore whether the TNF family gene signature affected the immunotherapy response of patients with CC, we first calculated and compared the 22 human immune cell subpopulations between the high- and low-risk groups. Notably, we found dendritic cells resting, macrophage M1, mast cells resting, plasma cells, T cells CD8, T cells follicular helper, and T cells regulatory were obviously up-regulated in the low-risk group compared with the high-risk group, while macrophage M0, mast cells activated, NK cells resting were significantly down-regulated in the low-risk group (**Figure 5A**), which were consistent with the results of functional enrichment analysis. Moreover, we also compared the expression levels of immune checkpoint molecules, including CD274 (PD-L1), CTLA4, LAG3, and PDCD1 (PD-1), between the high- and low-risk groups (**Figure 5B**). Interestingly, we found that these genes were upregulated in the low-risk group. Moreover, the low-risk group had a lower TIDE score than the high-risk group and was potentially more sensitive to anti-PD1 therapy (**Figures 5C, D**).

### Potential Regulatory Mechanisms of Genes in the TNF Family Gene Signature

**Figure 6:**
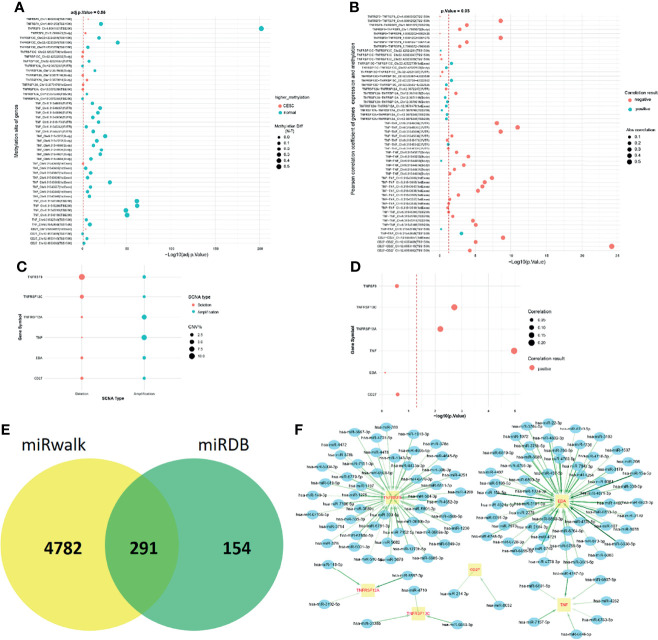
Potential regulatory mechanisms of genes in the TNF family gene signature. **(A)** Different biomarker methylation sites in normal and tumour tissues. The figure shows a total of five biomarkers and 49 different methylation sites. **(B)** Fifty-six genes are correlated with the mRNA expression levels of corresponding methylation sites, of which 29 pairs were significant. **(C)** Copy number variation of the six biomarkers. **(D)** Correlation between copy number variation and gene expression. **(E)** Venn diagrams for the EmiRwalk and miRDB databases. **(F)** Construction of miRNA-mRNA regulatory networks: the green arrow indicates the interaction, and the darker the colour, the stronger the interaction. Blue ellipses represent miRNAs and yellow rectangles represent target genes.

To further understand the reasons for the altered expression of these genes in the TNF family gene signature, we further observed the methylation levels and copy number variation of *CD27*, *EDA*, *TNF*, *TNFRSF12A*, *TNFRSF13C*, and *TNFRSF9*. For methylation analysis, we only found data for *CD27*, *TNF*, *TNFRSF12A*, *TNFRSF13C*, and *TNFRSF9*. Most methylation sites were differentially expressed in normal and patients with CC, and most differential methylation levels were negatively correlated with expression levels (**Figures 6A, B**). Copy number variants occurred in *CD27*, *EDA*, *TNF*, *TNFRSF12A*, *TNFRSF13C*, and *TNFRSF9* in patients with CC, where the copy number variation possibly affected their expression levels (**Figures 6C, D**). Finally, we predicted 291 target miRNAs of *CD27*, *EDA*, *TNF*, *TNFRSF12A*, *TNFRSF13C*, and *TNFRSF9* and constructed a miRNA-mRNA network (**Figure 6E**). We speculated that CD27 might be regulated by hsa-miR-214-3p and hsa-miR-8052 and that TNFRSF13C might be regulated by hsa-miR-4710, hsa-miR-3135b, and hsa-miR6848-5p (**Figure 6F**).

### Prognostic Significance of Genes in the TNF Family Gene Signature in Gynaecologic Tumours As Well As BRCA

**Figure 7:**
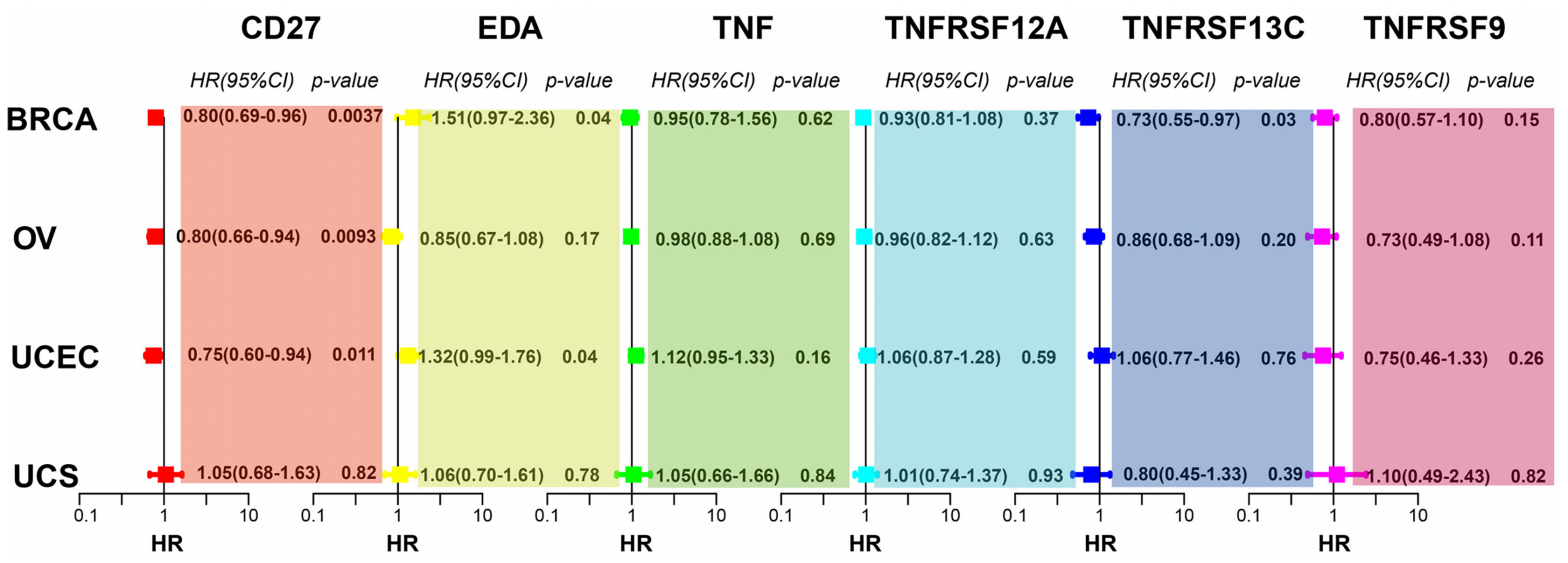
*CD27, EDA*, and *TNFRSF13C* are significantly correlated with BRCA. CD27 is significantly correlated with OV. *CD27* and *EDA* are significantly correlated with UCEC.

To investigate whether *CD27*, *EDA*, *TNF*, *TNFRSF12A*, *TNFRSF13C*, and *TNFRSF9* expression affects the prognosis of other gynaecologic tumours and BRCA, we performed univariate Cox regression analysis based on the overall survival information and expression of the aforementioned genes. *CD27* expression was associated with the overall survival of patients with BRCA, OV, UCEC, and UCS, while *EDA* was associated with UCEC prognosis and *TNFRSF13C* with BRCA prognosis (**Figure 7**).

### Validation of mRNA Expression of the TNF Family Gene Signature

**Figure 8:**
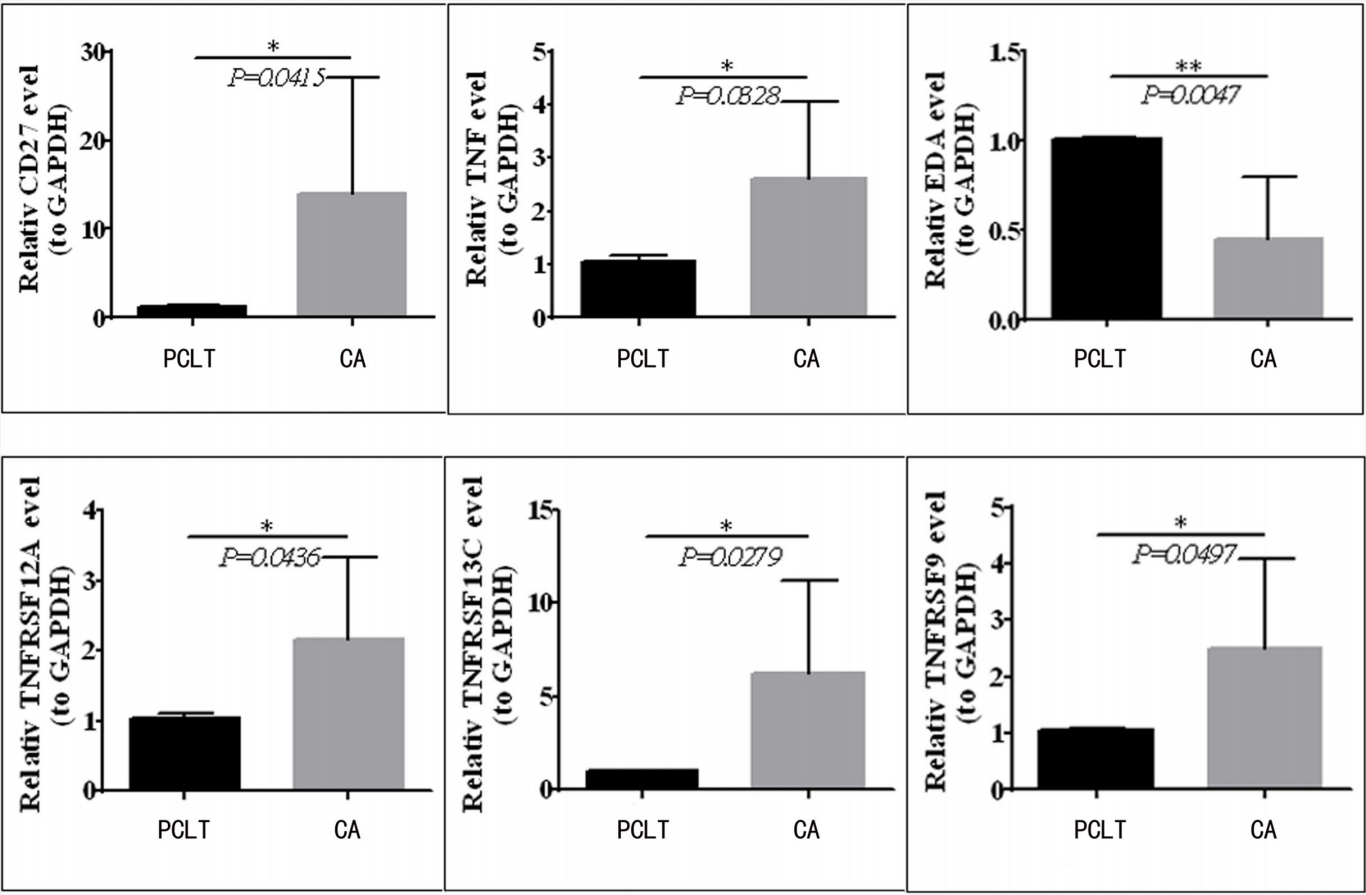
CD27, TNF, TNFRSF12A, TNFRSF13C, and TNFRSF9 expression is upregulated in CC tissues compared to paraneoplastic tissues, but EDA was downregulated in clinical samples.*means p < 0.05; **means p < 0.01.

To validate the mRNA expression of *CD27*, *EDA*, *TNF*, *TNFRSF12A*, *TNFRSF13C*, and *TNFRSF9*, we performed quantitative real-time PCR using clinical samples. The expression levels of *CD27*, *TNF*, *TNFRSF12A*, *TNFRSF13C*, and *TNFRSF9* were upregulated in the tissues from CC compared with those in paraneoplastic tissues; however, *EDA* expression was downregulated (**Figure 8**), which was consistent with the results of TCGA (**Figure 1C**).

## Discussion

Despite increasing human papilloma virus (HPV) vaccination and CC screening, the latter remains the fourth most common cancer worldwide (26). Although early-stage CC has an excellent prognosis, the 5-year overall survival rate for patients diagnosed as having stage III CC remains <40% (27). Currently, the first-line treatment for patients with early CC is radical hysterectomy and lymph node dissection, whereas radiotherapy and cisplatin-based chemotherapy are the optimal treatments for patients with advanced CC (28). Unfortunately, many patients with CC develop resistance to these drugs owing to the occurrence of adaptive chemotherapy resistance. The median overall survival of patients with advanced CC is 16.8 months, while the 5-year survival rate for all cases is only 68%, indicating that treatment is still not ideal (29). Therefore, elucidating the mechanisms leading to CC progression and identifying new prognostic markers are urgently needed.

In this study, using transcriptomic data of 306 patients with CC and 13 normal control samples, we screened 26 differentially expressed TNF family genes. A TNF family gene signature comprising *CD27*, *EDA*, *TNF*, *TNFRSF12A*, *TNFRSF13C*, and *TNFRSF9* was constructed based on univariate Cox, LASSO, and multivariate Cox regression analyses. Moreover, the KM and ROC curve analyses showed that the TNF family gene signature is a good prognostic marker for CC. Interestingly, we found that our gene signature was superior to other signatures. For example, the AUC of ROC curves for autophagy-related gene risk model predicting 1-, 3-, and 5-year overall survival was 0.678, 0.648, and 0.674, separately (30). The AUC of ROC curves for m6A RNA methylation regulator-related signature predicting 3- and 5-year overall survival was 0.67 and 0.72, separately (31). The AUC of ROC curves for hypoxia-related gene signature predicting 1-, 3-, and 5-year overall survival was 0.685, 0.683, and 0.683, separately (32). More importantly, univariate and multivariate Cox analyses showed that the TNF family gene signature was a useful prognostic factor independent of any other clinical factors in patients with CC. CD27, expressed by CD4, CD8 T lymphocytes, and NK cells, plays an important role in cancer immunotherapy (33). CD27 affects the prognosis of CC, and the higher its expression level, the better the prognosis (34). Likewise, CD27 is also particularly important for prognosis in multiple myeloma and clear cell renal cell carcinoma (35). EDA, a type II transmembrane protein whose receptor acts as a component of the Wnt/β-catenin signaling pathway, can affect the occurrence of colorectal cancer (36). Knockdown of the EDA receptor-associated adaptor protein has a tumour-suppressive effect on tongue squamous cell carcinoma (37). EDA receptors act as dependent receptors to control the progression of melanoma (38). However, the role of EDA in CC has not yet been reported. TNF is a proinflammatory and pro-apoptotic cytokine. TNF single-nucleotide variants (SNVs) have been found to be risk factors for the development of CC (39). The encoding protein tumour necrosis factor-α (TNF-α) of TNF can inhibit the survival of cancer cells and ultimately improve the prognosis of CC (40). TNFRSF12A, a member of the TNF receptor superfamily 12A, also known as FN14, CD266, and TWEAK, is the smallest member of the TNF superfamily receptor and contains a short cytoplasmic extinction domain (41). TNFRSF12A can be used as a prognostic marker for thyroid cancer (42) as it is associated with the progression of CC (43). TNFRSF13C (BAFFR or CD268), the receptor of BAFF, is an important regulator of B cell proliferation, development, and maturation (44). TNFRSF13C is associated with drug-resistant B cell malignancies and affects the prognosis and immunotherapy response of lung adenocarcinoma (45). TNFRSF9, also known as CD137, is found on activated T cells and plays an important role in tumour immunotherapy (46). CD137 is an important recognition factor for poor prognosis in patients with non-small cell lung cancer (47). In this study, we found that CD27 was associated with the overall survival of BRCA, OV, UCEC, and UCS patients, EDA was associated with the prognosis of UCEC, and TNFRSF13C was associated with BRCA prognosis. Therefore, we speculate that *CD27*, *EDA*, *TNF*, *TNFRSF12A*, *TNFRSF13C*, and *TNFRSF9* expression may play important roles in CC and may be good prognostic markers of CC.

To further study the mechanism by which the TNF family gene signature affects the prognosis of CC, we investigated the possible involvement of the TNF family gene signature in KEGG signaling pathways through GSEA. Notably, we found that the TGF-β signaling pathway was activated in the high-risk group. Therefore, we hypothesised that CD27, EDA, TNF, TNFRSF12A, TNFRSF13C, and TNFRSF9 may affect tumour prognosis mainly by regulating the TGF-β signaling pathway. The TGF-β signaling pathway is critical for the progression of CC (48, 49). For example, NSD2 can affect the metastasis of CC by regulating the TGF-β/TGF-βRI/SMAD signaling pathway (48). RHCG inhibits the development of CC by suppressing migration and inducing TGF-β 1-mediated apoptosis. On the other hand, genes in the high-risk group were mainly associated with immune-related pathways, and immune cells infiltrated more in the low-risk group compared with the high-risk group. It has been suggested that CD27 plays a key role in T-cell activation by providing a costimulatory signal (50). Moreover, CD27 improves expansion and differentiation of activated B cells into plasma cells in T cell-dependent B cell responses (51). Moreover, increasing evidence has revealed that TNF can promote tumor growth by recruiting neutrophils and macrophages, which benefit from inflammatory cytokines and chemokines (52). Furthermore, accumulation of preclinical data has suggested that TNF receptor family genes may neutralize tumor immunity by directly activating tumor-specific T cells or inhibiting dominant inhibitory mechanisms (53). TNFR family genes play a role in the intervention of CD28 to increase activation, survival and differentiation of effector and memory cells (54). Therefore, we speculated that better prognosis in the low-risk group might be related to immune activation. In addition, we analyzed the immunotherapy response differences between the high- and low-risk groups. The low-risk group exhibited significantly higher immune checkpoint molecular expression and lower TIDE score than the high-risk group. Additionally, subclass mapping analysis showed that the low-risk group was indeed more suitable for immunotherapy, which was consistent with the results of Wang et al. (55). Therefore, the TNF family gene signature not only affects the prognosis of CC but also affects the effect of immunotherapy on patients with CC. In other words, CD27, EDA, TNF, TNFRSF12A, TNFRSF13C, and TNFRSF9 expression may also affect the immunotherapy effect on tumours by regulating the expression of immune checkpoint molecules in the tumour microenvironment.

Finally, we found that the alterations in the mRNA expression of *CD27*, *EDA*, *TNF*, *TNFRSF12A*, *TNFRSF13C*, and *TNFRSF9* are caused by changes in gene methylation or copy number. Therefore, we not only propose a prognostic marker for CC but also provide a new reference for the molecular basis of CC occurrence and development. Notably, TCGA and GTEx differential analyses results are consistent. The qRT-PCR results of the clinical samples also showed that the mRNA expression of *CD27*, *TNF*, *TNFRSF12A*, *TNFRSF13C*, and *TNFRSF9* is upregulated in cancer tissues, except *EDA* whose mRNA expression is downregulated in cancer tissues. The qRT-PCR results further emphasise the significance of *CD27*, *EDA*, *TNF*, *TNFRSF12A*, *TNFRSF13C*, and *TNFRSF9* expression in CC. However, a higher expression of *CD27*, *TNFRSF13C*, and *TNFRSF9* and a lower expression of *EDA* in tumours indicated better prognosis. This may be due to the dual role of genes in tumour progression and prognosis. For example, Wu et al. found that *INPP4B* is an oncogene as well as a tumour suppressor gene in different tissue grades and clinical stages (56). Cao et al. also found that *CXCL11* is highly expressed in colon cancer tissues, and the higher the expression level in cancer tissues, the better the prognosis (57). Generally speaking, our study firstly found that the TNF family gene signature could predict the prognosis of patients with CC, and had stronger predictive power than other gene signatures. Meanwhile, we found that the TNF family gene signature might affect the immunotherapy of patients with CC, thus providing a basis for guiding the immunotherapy of CC. However, this study had some limitations. Even though bioinformatics tools are helpful to identify the interactions of hub genes, wet-lab experiments using actual tissue samples are warranted to validate the molecular mechanisms underlying CC progression. Therefore, in future, we will further investigate the involvement as well as the mechanism underlying the actions of these genes in CC.

## Conclusions

We established a TNF family gene signature for patients with CC based on *CD27, EDA, TNF, TNFRSF12A, TNFRSF13C*, and *TNFRSF9*, which could predict the prognosis and immunotherapy of patients with CC. Collectively, in this study, we present a potential and novel prognostic biomarker for CC.

## Data Availability Statement

The original contributions presented in the study are included in the article/**Supplementary Material**. Further inquiries can be directed to the corresponding author.

## Ethics Statement

The studies involving human participants were reviewed and approved by Ethics Review Committee of Suzhou Municipal Hospital (Suzhou, China). The patients/participants provided their written informed consent to participate in this study.

## Author Contributions

MYYM and JQ designed this study. YM, XZ and YX were responsible for data collection and preparation. YM, JY and YJ contributed to data analysis. YM wrote the manuscript. JQ reviewed the data and manuscript. All authors read and approved final version of the manuscript.

## Conflict of Interest

The authors declare that the research was conducted in the absence of any commercial or financial relationships that could be construed as a potential conflict of interest.

## Publisher’s Note

All claims expressed in this article are solely those of the authors and do not necessarily represent those of their affiliated organizations, or those of the publisher, the editors and the reviewers. Any product that may be evaluated in this article, or claim that may be made by its manufacturer, is not guaranteed or endorsed by the publisher.

## Supplementary Material

Supplementary Figure 1 Evaluating the efficiency of the TNF family gene signature in the testing set. **(A)** Time-dependent ROC curve evaluated the efficiency of the TNF family gene signature for predicting 1-, 3- and 5-year OS. **(B)** KM survival curve showed the prognostic value of the TNF family gene signature.

## Abbreviations

AUC, area under the curve; BRCA, breast cancer; CC, cervical cancer; cDNA, complementary DNA; DEGs, differentially expressed genes; HPV, human papilloma virus; EMT, epithelial-mesenchymal transition; GSEA, gene set enrichment analysis; GTEx, Genotype-tissue expression; KEGG, Kyoto encyclopedia of genes and genomes; KM, Kaplan-Meier; LASSO, least absolute shrinkage and selection operator; OV, ovarian cancer; PCA, principal component analysis; ROC, receiver operating characteristic; SNVs, single-nucleotide variants; TCGA, The Cancer Genome Atlas; TIDE, tumour immune dysfunction and exclusion; TNF, tumour necrosis factor; TRAIL, TNF-related apoptosis-inducing ligand; UCEC, uterine corpus endometrial carcinoma; UCS, uterine carcinosarcoma; UCSC, University of California Santa Cruz.
