# Supplementary figures and images for: Comprehensive Molecular Analyses of a TNF Family-Based Gene Signature as a Potentially Novel Prognostic Biomarker for Cervical Cancer
Source: Front Oncol. 2022 Mar 22;12:854615. doi: 10.3389/fonc.2022.854615 (PMC8980547; doi:10.3389/fonc.2022.854615)

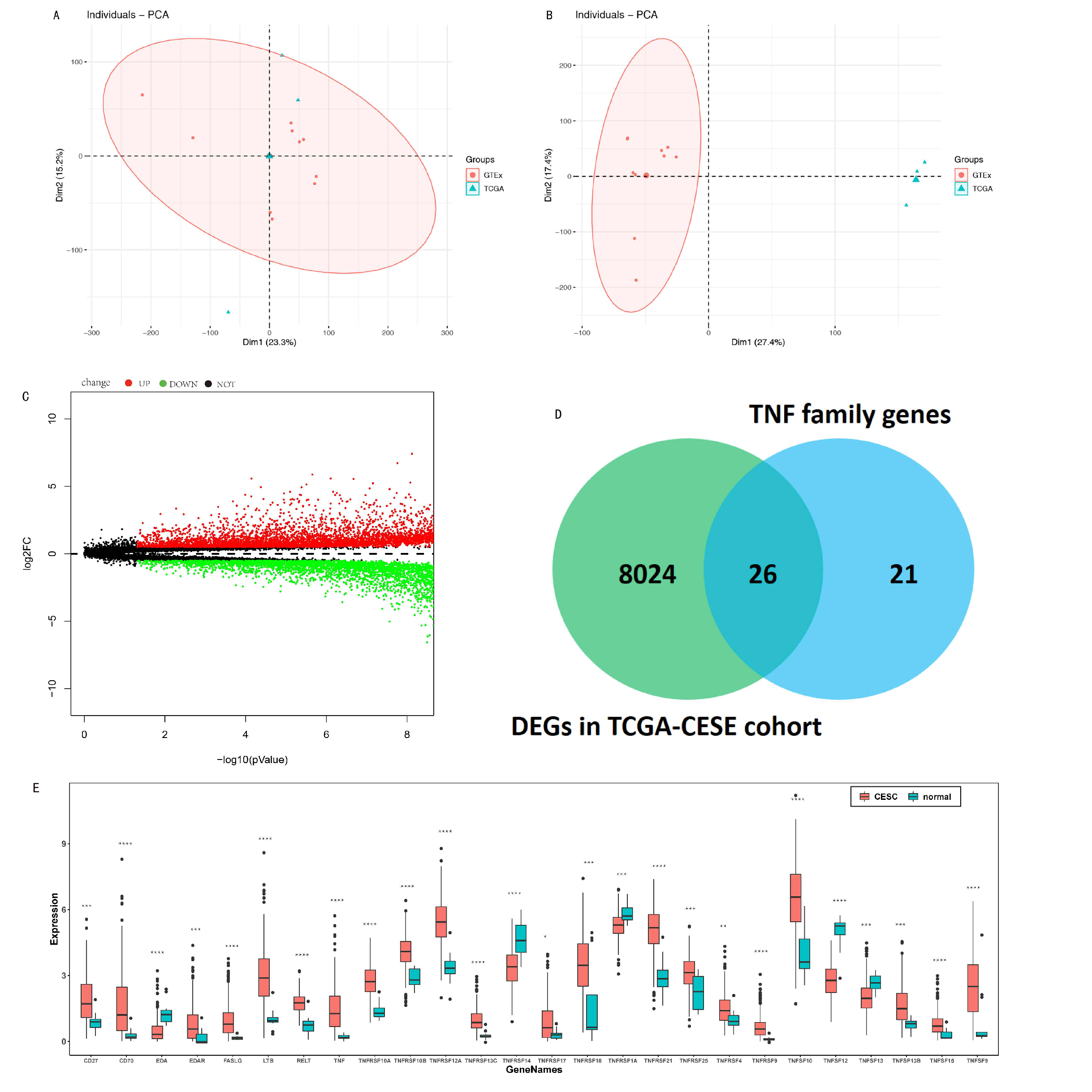

Supplement: Supplementary Figure 1 — Evaluating the efficiency of the TNF family gene signature in the testing set. (A) Time-dependent ROC curve evaluated the efficiency of the TNF family gene signature for predicting 1-, 3- and 5-year OS. (B) KM survival curve showed the prognostic value of the TNF family gene signature. [file Image_1.tif]
