# Supplementary material for: Comprehensive Molecular Analyses of a TNF Family-Based Gene Signature as a Potentially Novel Prognostic Biomarker for Cervical Cancer
Source: Front Oncol. 2022 Mar 22;12:854615. doi: 10.3389/fonc.2022.854615 (PMC8980547; doi:10.3389/fonc.2022.854615)
Supplement: Supplementary file 2 [file Table_1.docx]

Table S1 The primer sequences

| Gene | Sequence（5^，^→3^,^） | |
| --- | --- | --- |
| *CD27* | Forward Primer | CAGATGTGTGAGCCAGGAA |
| *CD27* | Reverse Primer | AGCGAAGGGTTTGGAAGAG |
| *EDA* | Forward Primer | CCGCAATAAAAGAAGCAAAAG |
| *EDA* | Reverse Primer | GCTGGCAAAGTCAGTGAAGTT |
| *TNF* | Forward Primer | TGCTCCTCACCCACACCAT |
| *TNF* | Reverse Primer | GGAAGACCCCTCCCAGATA |
| *TNFRSF12A* | Forward Primer | CTTTCTGGCTTTTTGGTCTGG |
| *TNFRSF12A* | Reverse Primer | GGGGGTGGTGAACTTCTCTCT |
| *TNFRSF9* | Forward Primer | TCCTGGTGGGTCTGGTGAG |
| *TNFRSF9* | Reverse Primer | GGGGTGGTTCCTGGGTCTT |
| *GAPDH* | Forward Primer | CCCATCACCATCTTCCAGG |
| *GAPDH* | Reverse Primer | CATCACGCCACAGTTTCCC |
